# Phenolic compounds in species of the Blechnaceae

**DOI:** 10.1111/plb.70116

**Published:** 2025-09-28

**Authors:** M. Ufland, M. Petersen

**Affiliations:** ^1^ Institut für Pharmazeutische Biologie und Biotechnologie Philipps‐Universität Marburg Marburg Germany

**Keywords:** Blechnic acid B, caffeoyl‐5‐*O*‐quinic acid, phenolic compounds, rosmarinic acid

## Abstract

- Rosmarinic acid and other caffeic and 4‐coumaric acid derivatives are widespread in land plants. These phenolic compounds contribute to the medicinal uses of plants. Ferns (Polypodiopsida), however, are still under‐investigated in terms of phenolic natural products and their biosyntheses.
- We analysed 32 fern species in the Blechnaceae, encompassing all three subfamilies, for content of caffeoyl‐5‐*O*‐quinic acid, rosmarinic acid and related compounds using LC/ESI‐MS/MS and HPLC. A compound previously unknown from ferns, but known in two species of Lamiaceae, was purified, its structure elucidated by NMR, and named blechnic acid B.
- While caffeoyl‐5‐*O*‐quinic acid was present in extracts from all investigated species, rosmarinic acid was only found in four species (*Doodia maxima*, *Lomariocycas tabularis*, *Neoblechnum brasiliense*, *Oceaniopteris ciliata)* belonging to superclade B of the Blechnoideae subfamily. Blechnic acid B, a novel compound in ferns, is composed of two moieties of isorinic acid (caffeoyl‐4′‐hydroxyphenyllactic acid). This lignan was only detected in *Neoblechnum brasiliense*, together with three related lignans having one or two 3,4‐dihydroxyphenyllactic acid moieties.
- Our study suggests that genes for biosynthesis of caffeoyl‐5‐*O*‐quinic acid were already established in ancestor species of the Blechnaceae. Blechnic acid B and its three derivatives might have formed through a selective coupling of the rosmarinic/isorinic acid monomers with the help of a dirigent protein.

## Graphical abstract

The occurrence of caffeic acid derivatives in 32 species of Blechnaceae revealed differences in the presence of rosmarinic acid and blechnic acid B, a novel compound in ferns.

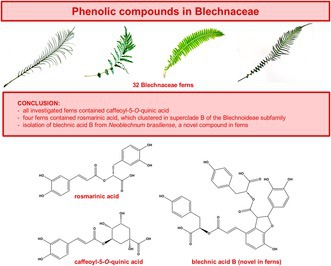

## INTRODUCTION

The Blechnaceae family are leptosporangiate ferns in the order Polypodiales, suborder Aspleniineae (Eupolypods II) (Nitta *et al*. 2022). Members of the Blechnaceae are distributed across the globe, with the Neotropics and Australasia/Oceania as major centers (Kramer & Green 1990; de Gasper *et al*. 2017). Recent molecular data suggest that the 200–250 species are divided among 24 genera in three subfamilies, with Blechnoideae being the largest subfamily (de Gasper *et al*. 2016; de Gasper *et al*. 2017). Perrie *et al*. (2014) showed that the genus *Blechnum* is polyphyletic and described *Telmatoblechnum* as a new genus within the subfamily Stenochlaenoideae (Super‐Stenochlaena). de Gasper *et al*. (2016), de Gasper *et al*. (2017) introduced *Austroblechnum*, *Cleistoblechnum*, *Cranfilla*, *Icarus*, *Neoblechnum*, and *Oceaniopteris* as new genera, and re‐introduced the old genera *Parablechnum*, *Struthiopteris*, *Blechnopsis*, *Parablechnum*, *Lomaridium*, *Lomaria*, and *Diploblechnum* in order to achieve the monophyletic genus *Blechnum* s.s. These authors also defined two superclades within the subfamily Blechnoideae consisting of superclade A (*Icarus*, *Cranfilla*, *Blechnum*, and *Austroblechnum*) and superclade B (*Parablechnum*, *Doodia*, *Neoblechnum*, *Oceaniopteris*, *Lomariocycas*, and *Diploblechnum*).

The current taxonomy of pteridophytes was published by the Pteridophyte Phylogeny Group (PPG I 2016). Pteridophytes are composed of two classes: the lycophytes (Lycopodiopsida) and the ferns (Polypodiopsida). One subclass (out of four) of the Polypodiopsida is the leptosporangiate ferns (Polypodiidae), with seven orders (Osmundales, Hymenophyllales, Gleicheniales, Schizaeales, Salviniales, Cyatheales, Polypodiales). The family Blechnaceae belongs to the order Polypodiales, which contains 26 families in six sub‐orders.

Ferns are rich in specialized metabolites, such as flavonoids, terpenoids, steroids, and polyphenols (Cao *et al*. 2017; Waswa *et al*. 2022). The abundance of natural substances led to the use of plants in the family Blechnaceae in Traditional Chinese Medicine, Traditional Indian Medicine, and Chilean Traditional Medicine. Traditional medicinal uses of blechnaceous ferns include for skin disorders, bladder infections, pulmonary diseases, and gastrointestinal disorders (Waswa *et al*. 2022).

**Fig. 1:**
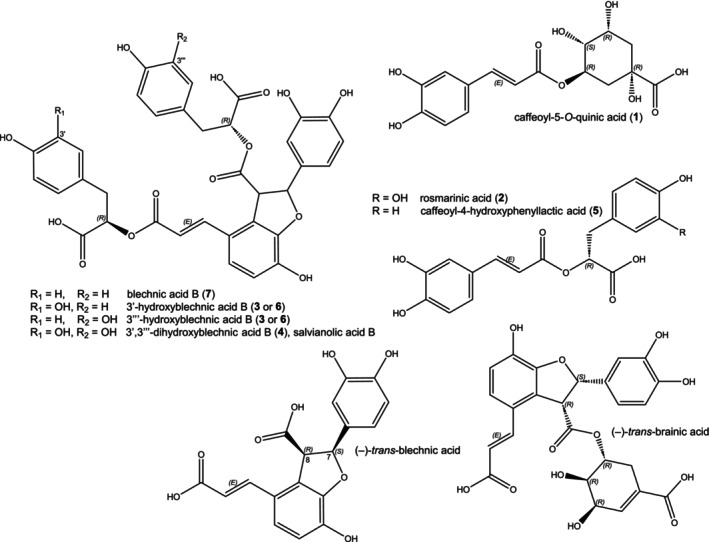
Examples of phenolic compounds in Blechnaceae ferns. Stereocenters of caffeoyl‐5‐*O*‐quinic acid shown for l‐quinic acid according to Abrankó *et al*. (2017).

Rosmarinic acid (RA, **2**; Fig. 1), an ester of caffeic acid and ( *r*)‐3,4‐dihydroxyphenyllactic acid, was first discovered in rosemary (Scarpati & Oriente 1958) and is distributed primarily in the Boraginaceae and subfamily Nepetoideae of the Lamiaceae. Furthermore, RA was also identified in hornworts, e.g., *Anthoceros agrestis* (Takeda *et al*. 1990), Chloranthaceae (*Sarcandra glabra*; *Chloranthus officinalis*; Petersen *et al*. 2009; Zhu *et al*. 2008; Bömeke & Petersen 2025), and various monocots and dicots (Petersen *et al*. 2009). Also, some ferns have been described as containing RA, e.g., *Neoblechnum brasiliense* (formerly *Blechnum brasiliense*, Blechnaceae, Polypodiales; Harborne 1966; Bohm 1968; Andrade *et al*. 2016), *Adiantum capillus‐veneris* (Pteridaceae, Polypodiales) (Zeb & Ullah 2017), *Azolla filiculoides* (Salviniaceae, Salviniales) (Carballo‐Sanchez *et al*. 2022), and the edible fern *Diplazium esculentum* (Athyriaceae, Polypodiales) (Kongsung *et al*. 2024).

Another caffeic acid derivative in members of the Blechnaceae is caffeoyl‐5‐*O*‐quinic acid (or 5‐caffeoylquinic acid, 5‐CQA, **1**; e.g., Bohm 1968) (Fig. 1), an ester of caffeic acid with the 5‐hydroxy group of l‐quinic acid, which was first described by Robiquet and Boutron in 1837 (Sondheimer 1964). The nomenclature in our report follows Abrankó & Clifford (2017), as the numbering here is based on l‐quinic acid rather than the ester, as esterification would invert the ring numbering and the chirality of the stereocenters in C1 and C4, leading to misunderstandings. 5‐CQA is found in many species throughout the plant kingdom and is abundant in the orders Apiales, Asterales, and Dipsacales (Petersen *et al*. 2009). Several reviews have summarized the occurrence and biological activities of 5‐CQA and related compounds (Clifford 1999, 2000; Clifford *et al*. 2017). 5‐CQA and its isomers have also been described from fern species of various families (Dion *et al*. 2015; Zeb & Ullah 2017; Farràs *et al*. 2022; Salazar‐Chacón *et al*. 2024).

Blechnic acid (Fig. 1), a lignan formed from two caffeic acid moieties, was first described by Bohm (1968) and structurally elucidated by Wada *et al*. (1992). Besides blechnic acid, 7‐*epi*‐blechnic acid, 8‐*epi*‐blechnic acid, and brainic acid (Fig. 1) were isolated from blechnaceous ferns (Wada *et al*. 1992). Labeling experiments have been performed with *Blechnum spicant* and resulted in ^13^C‐labelled (−)‐*cis*‐blechnic acid, (−)‐*trans*‐blechnic acid, and (−)‐*trans*‐brainic acid, showing that they are derived from l‐phenylalanine (Davin *et al*. 2003). Further congeners of blechnic acid have been identified, e.g., salvianolic acid B from *Salvia miltiorrhiza* (Ai & Li 1988), which is the same compound as lithospermic acid B (Watzke *et al*. 2006). Sebestenoids C and D are further congeners of blechnic acid isolated from *Cordia sebestena* (Boraginaceae) (Dai *et al*. 2010).

This study focuses on phenolic natural products, with particular focus on caffeic acid derivatives (see Fig. 1) and their distribution within the family Blechnaceae.

## MATERIAL AND METHODS

### Plant material

Fern fronds were obtained from various botanical gardens across Germany and transported via mail wrapped in moist paper towels. Fern fronds were photographed upon arrival and compared with images of previously identified species (https://powo.science.kew.org). The fresh pinnae were collected in the laboratory and freeze‐dried overnight. The lyophilized pinnae were stored at −80°C. Sources and accession numbers for all plants analysed in this study are listed in Table S1.

### Extraction of phenolic compounds

The lyophilized pinnae were pulverized in a mortar and pestle, and 100 mg transferred into a Precellys^®^ hard tissue grinding MK28‐R vial with 1 mL 70% ethanol. The pinnae were homogenized for 180 s at 5000 rpm in a benchtop homogenizer (Minilys^®^), followed by ultrasonication for 10 min at 70°C. This process of homogenization and ultrasonication was repeated. The cell residues were sedimented by centrifugation for 10 min at 17,000×*g*. The supernatant was transferred to a fresh tube and 1 mL 70% ethanol added to the residue, vortexed for 1 min, and again centrifuged for 10 min at 17,000×*g*. The supernatant was combined with the first extract. An aliquot of the extract was diluted 1:10 with 50% methanol, 0.01% phosphoric acid, and centrifuged for 10 min at 17,000×*g* prior to HPLC analysis. Each extraction was performed in triplicate.

### Qualitative and quantitative analysis of phenolic compounds

For qualitative analysis LC/ESI‐MS/MS (HPLC: Agilent 1260 series, column: Multospher 120 RP 18, 5 μm, length 250 mm, ∅ 2 mm; A: 0.1% formic acid in water, B: 0.1% formic acid in acetonitrile; 0–40 min 5% B → 100% B, 40–45 min 100% B, 45–45.10 min 100% B → 5% B, 45.10–55 min 5% B; flow rate: 0.5 mL min^−1^; mass spectrometer: micrOTOF‐Q III with ESI‐source (Bruker Daltronics, Billerica, MA, USA), calibration with 5 mM sodium formate, negative mode) was performed against external standards of RA and 5‐CQA.

Quantitative analysis was performed on a Chromaster HPLC system (Hypersil ODS C18, 5 μm, length 250 mm, ∅ 4 mm; 35% methanol, 0.01% H_3_PO_4_ for isocratic elution, 1 mL min^−1^, 35°C) against external quantitative standards of RA, 5‐CQA, and blechnic acid B (BAB) (see below for isolation).

### Extraction of pinnae for isolation of blechnic acid B from *Neoblechnum brasiliense*

A subsample of 13.0 g freeze‐dried pinnae from *N. brasiliense* (Desv.) de Gasper *et al*. (2016), obtained from the Botanical Garden of the Philipps‐Universität Marburg (see Table S1 for accession), was pulverized with a mortar and pestle and transferred into a beaker. 250 mL 70% ethanol was added, and the pinnae homogenized with an Ultra‐Turrax^®^ T25 basic for 5 min at 22,000 rpm, and ultrasonicated at 70°C for 10 min. Homogenization and ultrasonication were repeated. The extract was freed of particles by centrifugation for 10 min at 10,000×*g* and filtered through MN 615 paper filters (Macherey‐Nagel, Düren, Germany). The solvent was evaporated with a rotary evaporator, resulting in 2.33 g crude dry extract.

### Isolation of blechnic acid B from pinnae of *Neoblechnum brasiliense*

The crude dry extract was redissolved in 20 mL 10% acetonitrile, 0.1% formic acid and centrifuged at 10,000×*g* until the extract cleared. In steps of 1.8 mL, the extract was then successively separated on a puriFlash^®^ 5.250P (Advion Interchim, Montluçon Cedex, France) with a gradient starting at 10% acetonitrile, 0.1% formic acid, increasing to 30% acetonitrile, 0.1% formic acid over 40 min (flow rate 20 mL min^−1^; Kinetex^®^ 5 μm C18 100 Å LC column, length 150 mm, ∅ 21.2 mm; detection at 333 nm). The novel compound blechnic acid B (BAB) with a retention time of 36.25 min was collected. The solvent of the collected peaks was evaporated by lyophilization, resulting in 15.5 mg BAB as an amorphous brown powder.

### *In‐silico* modelling of LC/ESI‐MS/MS data in SIRIUS and structure elucidation

In LC/ESI‐MS/MS analysis (see above), the novel compound (BAB, **7**) eluted at a retention time of 16.5 min and had a *m/z* [M‐H]^−^ of 685.1525. LC/ESI‐MS/MS data were transferred into SIRIUS and, with the help of SIRIUS (Dührkop *et al*. 2019) and CSI:FingerID (Dührkop *et al*. 2015), a putative fragment of the isolated compound was shown to be blechnic acid. 1‐D NMR spectra (^1^H (500 MHz), ^13^C (126 MHz)) and 2‐D spectra (^1^H‐^1^H COSY, HSQC, HMBC) were recorded in CD_3_OD, which served also as reference (δ_H_ = 3.31 ppm, δ_C_ = 49.00 ppm; Gottlieb *et al*. 1997) on a Jeol ECA500 spectrometer.

### Hydrolysis of blechnic acid B and detection of (*R*)‐4‐hydroxyphenyllactic acid

A total of 1 mg isolated BAB (**7**) was dissolved in 5 mL 0.1 M NaOH and hydrolyzed for 5 h at 60°C. The lysate was acidified with 1 mL 6 N HCl and successively extracted four times with 10 mL ethyl acetate each time. The ethyl acetate extract was dried with Na_2_SO_4_, the solvent evaporated, and the residue redissolved in 500 μL 96% ethanol. Chiral analysis was performed by HPLC on a Thermo Spectra System (column: Chiralcel^®^ OD, length 250 mm, ∅ 4.6 mm; Daicel Chemical Industries, Tokyo, Japan). The lysate (10 μL) and commercial standards of racemic 4‐hydroxyphenyllactic acid (100 nmol), (*s*)‐4‐hydroxyphenyllactic acid (50 nmol), were isocratically separated (92.5% *n*‐hexane, 7.5% isopropanol, 0.1% trifluoroacetic acid, flow rate 1 mL min^−1^, detection at 280 nm).

## RESULTS

### Extraction of ferns in the Blechnaceae for determination of rosmarinic acid and caffeoyl‐5‐*O*‐quinic acid

The occurrence of RA (**2**) and 5‐CQA (**1**) in members of the Blechnaceae has been reported previously (Harborne 1966; Bohm 1968; Szabo *et al*. 1993; Andrade *et al*. 2016). In addition, further hydroxycinnamic acid derivatives have been identified, e.g., blechnic acid and related compounds (see Fig. 1), as well as brainic acid (Wada *et al*. 1992). Since we study biosynthesis of RA and 5‐CQA in plant species across the plant kingdom, we first wanted to know which Blechnaceae species contain these molecules and extracted Blechnaceae species collected from different botanical gardens for phytochemical analysis of hydroxycinnamate compounds. Besides the known compounds RA (**2**) and 5‐CQA (**1**), there was a prominent peak in extracts of *N. brasiliense* that was collected for structure determination (see below).

### Structure elucidation of blechnic acid B (**7**)

The fractionation of an ethanolic extract from lyophilized *N. brasiliense* pinnae (13.0 g) resulted in 15.5 mg of an unknown compound, later named blechnic acid B (BAB, **7**), which was subjected to LC–MS (see below) and NMR analyses. LC–MS indicated a [M‐H]^−^ *m/z* of 685.1523, corresponding to a molecular formula of C_36_H_30_O_14_. Analysis by SIRIUS (Dührkop *et al*. 2019) and CSI:FingerID (Dührkop *et al*. 2015) revealed that the novel compound might contain a blechnic acid moiety.

**Fig. 2:**
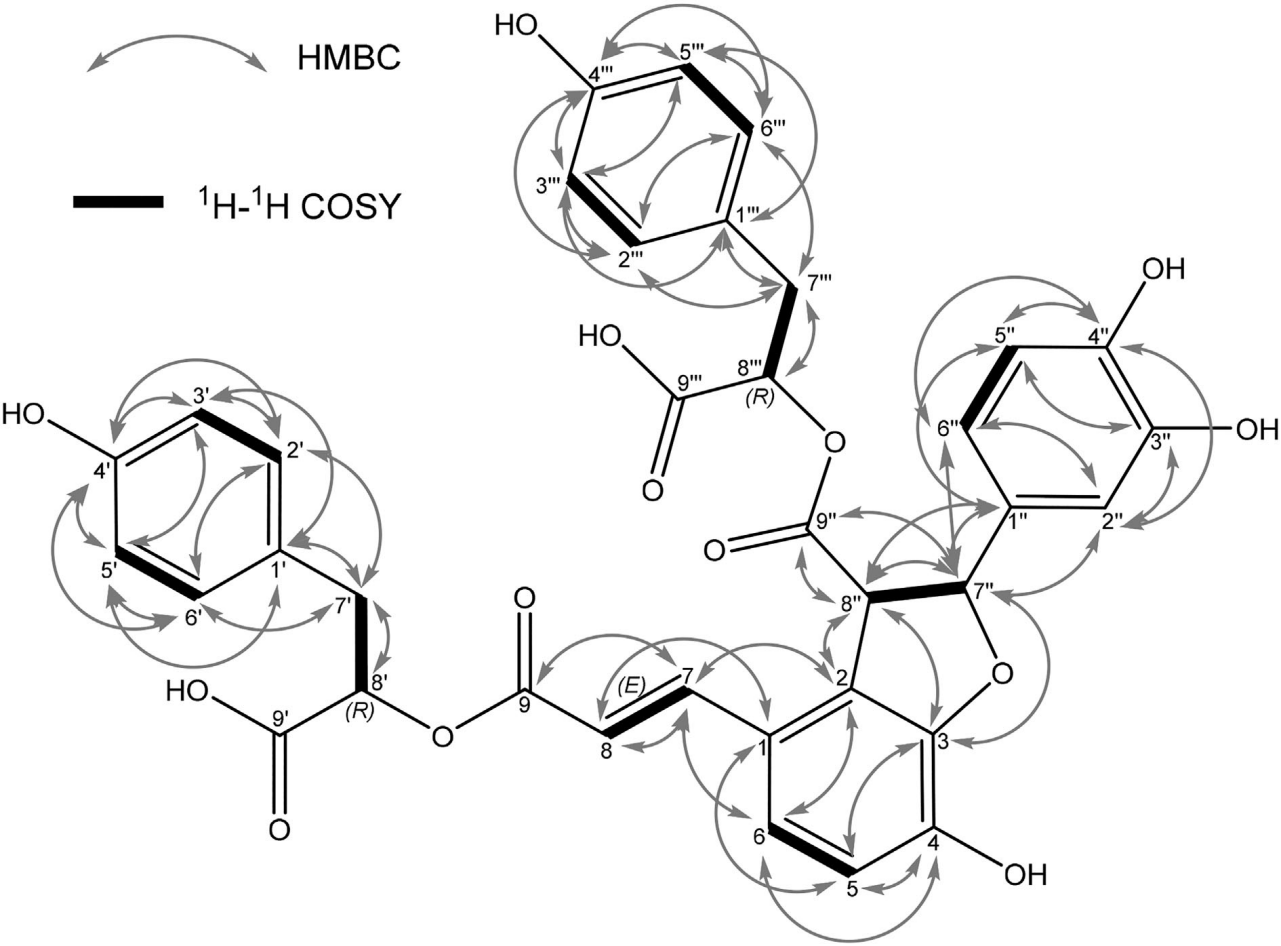
^1^H‐^1^H COSY and HMBC correlations of blechnic acid B (**7**) from *Neoblechnum brasiliense*.

Results of the ^1^H‐, ^13^C‐ and HSQC‐NMR evaluation are depicted in Table S2. All ^1^H‐^1^H‐COSY and HMBC correlations are shown in Fig. 2. The evaluation of the downfield of the ^1^H‐NMR showed two identical 1,4‐di‐substituted, one 1,2,3,4‐tetra‐substituted, and one 1,3,4‐tri‐substituted benzene. The observed coupling constant of H7″‐H8″ is 5.0 Hz, which indicates *trans*‐configured vicinal stereocenters. Comparing the coupling constant to similar compounds (vicinal C atoms in *cis*: 9.4 Hz, (−)‐*trans* blechnic acid (Davin *et al*. 2003); 9.0 Hz, 7,8‐*epi*‐blechnic acid (Jiang *et al*. 2022); vicinal C atoms in *trans*: 5.0 Hz, sebestenoid E (Jiang *et al*. 2022)) further supports this assumption. Unfortunately, our attempts to determine the absolute stereochemistry at H7″ and H8″ failed. No HMBC signal between C9‐H8′ and C9″‐H8″′ was seen. An assignment, which system (1′‐9′ or 1″′‐9″′) is esterified with C9/C9″, is therefore not possible, although it is not essential, since the 1′‐9′ and the 1″′‐9″′ systems are chemically identical and therefore interchangeable. To determine the absolute stereochemistry of the two 4‐hydroxyphenyllactic acid moieties, BAB (**7**) was hydrolyzed and separated on a chiral column resulting in both 4‐hydroxyphenyllactic acid moieties being (*r*)‐configured (Fig. S1). The coupling constant for H7‐H8 is 16.0 Hz, indicating that the double bond is in (*E*)‐configuration. The summarized data lead to structure (**7**), shown in Fig. 1, named blechnic acid B (BAB).

### Detection and identification of phenolic compounds by LC/ESI‐MS/MS

**Fig. 3:**
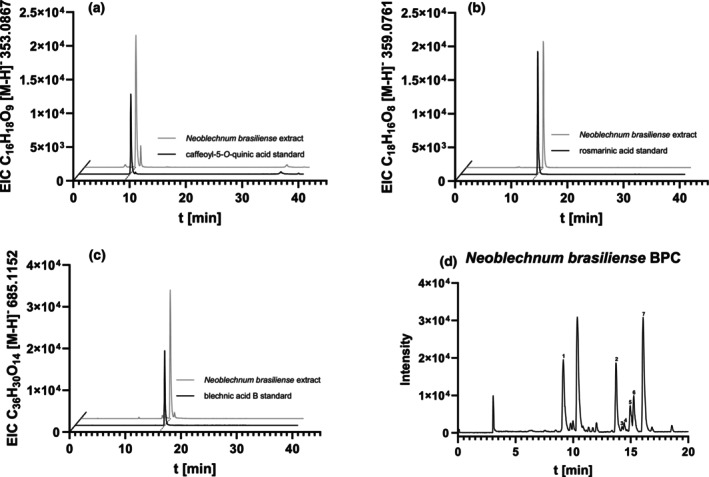
Extracted ion chromatograms of (a) caffeoyl‐5‐*O*‐quinic acid (**1**), (b) rosmarinic acid (**2**) and (c) blechnic acid B (**7**) in extracts from *Neoblechnum brasiliense*, (d) base peak chromatogram of the *N. brasiliense* extract. Peaks of identified compounds are numbered.

All extracts from Blechnaceae ferns were analysed for the occurrence of 5‐CQA (**1**), RA (**2**), and isorinic acid (caffeoyl‐4′‐hydroxyphenyllactic acid, **5** (Satake *et al*. 1999)) using authentic external standards. Other phenolic compounds were tentatively identified according to their LC/ESI‐MS/MS fractionation patterns. As an example, the LC/ESI‐MS/MS data for the extract of *N. brasiliense* are summarized in Table 1. Base peak and extracted ion chromatograms of the extract from *N. brasiliense* are shown in Fig. 3.

**Table 1:** Summary of identified phenolic compounds in *Neoblechnum brasiliense* and resulting LC/ESI‐MS and LC/ESI‐MS/MS data; *m/z* calculated with Compass Data Analaysis.

com‐pound no.	substance	molecular formula	retention time [min]	measured [M‐H]^−^ (*m/z*)	calculated [M‐H]^−^ (*m/z*)	LC/ESI‐MS/MS *m/z* (% base peak)
**1**	caffeoyl‐5‐*O*‐quinic acid^a^	C_16_H_18_O_9_	9.8	353.0852	353.0867	MS2[353]: 191 (100)
**2**	rosmarinic acid^a^	C_18_H_16_O_8_	14.3	359.0748	359.0761	MS2[359]: 161 (100), 179 (44), 197 (28)
**3**	3′‐hydroxyblechnic acid B^c^ *or* 3″‐hydroxyblechnic acid B^c^	C_36_H_30_O_15_	14.7	701.1710	701.1501	MS2[701]: 295 (97), 321 (100), 339 (46), 486 (14), 503 (36), 519 (3)
**4**	3′,3″‐dihydroxyblechnic acid B^c^	C_36_H_30_O_16_	14.9	717.1675	717.1450	MS2[717]: 295 (17), 321 (100), 339 (38), 519 (23)
**5**	caffeoyl‐4′‐hydroxyphenyllactic acid (isorinic acid)^a^	C_18_H_16_O_7_	15.5	343.0794	343.0812	MS2[343]: 135 (19), 161 (100), 181 (38)
**6**	3′‐hydroxyblechnic acid B^c^ *or* 3″‐hydroxyblechnic acid B^c^	C_36_H_30_O_15_	15.8	701.1449	701.1501	MS2[701]: 295 (18), 321 (100), 339 (47), 503 (16), 519 (8)
**7**	blechnic acid B^b^	C_36_H_30_O_14_	16.6	685.1523	685.1152	MS2[685]: 295 (14), 321 (100), 339 (50), 503 (21)

^a^ Compared with reference substance.

^b^ Purified and elucidated via NMR.

^c^ Proposed from LC/ESI‐MS/MS data.

5‐CQA (**1**) has a mass peak at *m/z* 353.0852; the MS2 signal *m/z* 191 relates to a quinate fragment, while the caffeate fragment is not visible, neither in the extract nor in the standard. This is in accordance with Clifford *et al*. (2003), where 5‐CQA also produced a very low caffeate fragment (5% base peak intensity). The other regioisomers, 3‐CQA and 4‐CQA, produced caffeate fragments from 50% to 68% of the base peak intensity, with 4‐CQA additionally differing in having *m/z* 173 as the base peak. 5‐CQA (**1**) and its fragment could be detected in all examined blechnaceous ferns. Other caffeoylquinate peaks were detected, but only in traces.

The RA (**2**) base peak is at *m/z* 359.0748 and the resulting MS2 signals at *m/z* 179 and *m/z* 197 are the caffeate and 3,4‐dihydroxyphenyllactate fragments, respectively. RA and its fragments are found in *Doodia maxima*, *Oceaniopteris ciliata*, *N. brasiliense*, and *Lomariocycas tabularis*.

Caffeoyl‐4′‐hydroxyphenyllactic acid (isorinic acid; **5**) shows a base peak at *m/z* 343.0794 and the MS2 signal *m/z* 181 indicates the 4‐hydroxyphenyllactate fragment. Caffeoyl‐4′‐hydroxyphenyllactic acid (**5**) and its fragment could be detected in *O. ciliata*, *N. brasiliense* and *L. tabularis*, but not in *D. maxima*.

BAB (**7**) had the longest retention time and a mass peak at *m/z* 685.1523. The MS2 fragmentation pattern of BAB results in the following fragments: *m/z* 503 relates to BAB that lost one 4‐hydroxyphenyllactic acid moiety (−182). A further loss of a 4‐hydroxyphenyllactic acid moiety (−182) results in the fragment *m/z* 321.

Compounds **3** and **6** (*m/z* 701.1449) show a fragmentation pattern identical to compound **7**, with an additional fragment at *m/z* 519, which indicates the fragment *m/z* 503 mentioned above plus one additional oxygen. Observing both *m/z* 503 and 519 leads to the conclusion that compounds **3** and **6** are both BAB (**7**) analogs, where one of the 4‐hydroxyphenyllactic acid moieties carries a second OH‐group, tentatively in position 3. We suggest position 3 because compounds **3** and **6** appear to be lignans formed from an isorinic acid (**5**) and a rosmarinic acid (**2**), that has the mentioned second OH‐group in position 3. However, it is not possible to clearly assign whether compounds **3** or **6** are 3′‐hydroxyblechnic acid B and 3″′‐hydroxyblechnic acid B, respectively, or a diastereomer of one of these compounds. The presence of only two of these compounds, together with the fact that only one compound with the mass of blechnic acid B (**7**) and 3′,3″′‐dihydroxyblechnic acid B (**4**) is present in the extract of *N. brasiliense*, suggests that blechnic acid B and its derivatives all have the same configuration, resulting in both 3′‐hydroxyblechnic acid B and 3″′‐hydroxyblechnic B acid being present in the extract. Moreover, it is possible that these four lignans may originate from the same biosynthetic pathway.

The fragmentation pattern of compound **4** is identical to that of compound **7**, but *m/z* 503 is missing. Thus, both 4‐hydroxyphenyllactic acid moieties carry a second OH‐group in position 3, as this lignan appears to be formed from two rosmarinic acid moieties (**2**). Compound **4** thus presumably is 3′,3″′‐dihydroxyblechnic acid B. BAB (**7**) and its derivatives **3**, **4** and **6** were only found in *N. brasiliense* among all investigated fern species.

### Quantification of phenolic compounds in species of the Blechnaceae

Out of the 24 genera in the Blechnaceae (de Gasper *et al*. 2016), we analysed 32 species from 11 genera. Out of the three subfamilies, Blechnoideae (18 genera, ~233 species), Woodwardioideae (3 genera, ~15 species), and Stenochlaenoideae (3 genera, ~12 species), at least one species each was tested. All analysed Blechnaceae members in this study contained 5‐CQA (**1**), ranging from 0.01% dry weight in *Doodia heterophylla* to 1.78% in *Blechnum gracile*. RA (**2**) was only detected in the Blechnoideae subfamily in four analysed species, all belonging to the superclade B according to de Gasper *et al*. (2017), at levels from 0.07% in *L. tabularis* to 0.27% in *N. brasiliense*. BAB (**7**) was only found in *N. brasiliense* (0.68% dry weight). All analysed species with their phenolic content are summarized in Table 2. The occurrence of phenolic acids in the different subfamilies of the Blechnaceae is described in more detail below.

**Table 2:** Quantification of rosmarinic acid (RA, **1**), caffeoyl‐5‐*O*‐quinic acid (5‐CQA, **2**), and blechnic acid B (BAB, **7**) in ethanolic extracts from pinnae of species of the family Blechnaceae.

species (according to Field (2020); de Gasper *et al*. (2016))	5‐CQA [% of DW]	RA [% of DW]	BAB [% of DW]
Blechnoideae – superclade B			
*Parablechnum loxense*	1.28 ± 0.04	n.d.	n.d.
*Parablechnum chilense*	0.14 ± 0.01	n.d.	n.d.
*Parablechnum minus*	0.02 ± 0.01	n.d.	n.d.
*Parablechnum cordatum*	0.04 ± 0.00	n.d.	n.d.
*Parablechnum novae‐zelandiae*	0.07 ± 0.00	n.d.	n.d.
*Doodia maxima*	0.26 ± 0.02	0.07 ± 0.01	n.d.
*Doodia dives*	0.32 ± 0.01	n.d.	n.d.
*Doodia heterophylla*	0.01 ± 0.01	n.d.	n.d.
*Doodia media*	0.29 ± 0.01	n.d.	n.d.
*Doodia aspera*	0.15 ± 0.02	n.d.	n.d.
*Oceaniopteris gibba* [Marburg]	0.21 ± 0.00	n.d.	n.d.
*Oceaniopteris gibba* [Frankfurt]	0.45 ± 0.01	n.d.	n.d.
*Oceaniopteris ciliata*	0.17 ± 0.00	0.11 ± 0.00	n.d.
*Neoblechnum brasiliense*	0.60 ± 0.03	0.27 ± 0.00	0.68 ± 0.06
*Lomariocycas tabularis*	0.11 ± 0.00	0.07 ± 0.01	n.d.
Blechnoideae – superclade A			
*Austroblechnum penna‐marina* subsp. *alpina*	0.16 ± 0.02	n.d.	n.d.
*Austroblechnum penna‐marina*	0.40 ± 0.01	n.d.	n.d.
*Blechnum occidentale*	0.87 ± 0.02	n.d.	n.d.
*Blechnum gracile*	1.78 ± 0.04	n.d.	n.d.
*Blechnum polypodioides*	0.17 ± 0.01	n.d.	n.d.
*Blechnum punctulatum*	0.09 ± 0.01	n.d.	n.d.
*Blechnum* cf. *punctulatum*	0.60 ± 0.02	n.d.	n.d.
*Blechnum appendiculatum*	0.60 ± 0.02	n.d.	n.d.
*Blechnum auriculatum*	1.20 ± 0.01	n.d.	n.d.
*Blechnum punctulatum* . var. *atherstonii*	0.54 ± 0.03	n.d.	n.d.
Blechnoideae – further genera			
*Lomaria discolor*	1.27 ± 0.03	n.d.	n.d.
*Struthiopteris spicant*	0.68 ± 0.01	n.d.	n.d.
Stenochlaenoideae			
*Stenochlaena tenuifolia*	0.83 ± 0.06	n.d.	n.d.
Woodwardioideae			
*Woodwardia radicans*	0.41 ± 0.06	n.d.	n.d.
*Woodwardia unigemmata*	0.72 ± 0.04	n.d.	n.d.
*Woodwardia fimbriata*	0.66 ± 0.08	n.d.	n.d.
*Woodwardia orientalis*	0.65 ± 0.03	n.d.	n.d.
*Woodwardia prolifera*	0.79 ± 0.08	n.d.	n.d.

Quantification was performed with HPLC, occurrence was verified by LC/ESI‐MS/MS. The content was measured in three separate extractions ± SD. For authorities and plant sources, see Table S1.

DW, dry weight; n.d., not detected.

*Subfamily Blechnoideae*: Superclade A as defined by de Gasper *et al*. (2017) contains four genera (~77 species), two of which were analysed by us. Two subspecies of *Austroblechnum penna‐marina*, out of a total of 39 species in *Austroblechnum*, were analysed: *A. penna‐marina* subsp. *alpina* (0.16% 5‐CQA) and *A. penna‐marina* (0.40% 5‐CQA). Out of the total of 25 species from the genus *Blechnum* s.s. eight species were analysed. Their 5‐CQA content ranged from 0.09% in *B. punctulatum* to 1.78% in *B. gracile*. RA (**2**) could not be detected in members of this superclade A.

Superclade B, as defined by de Gasper *et al*. (2017), encompasses six genera with ~118 species. In the genus *Parablechnum* (five analysed out of a total of 65 species) the content of 5‐CQA ranged from 0.02% to 1.28% dry weight, with *P. loxense* having the highest and *P. minus* having the lowest content. The content in genus *Doodia* (five examined out of a total of 19 species) ranged from 0.01% to 0.32% dry weight, with *D. dives* having the highest content of 5‐CQA and *D. heterophylla* having the lowest. *D. maxima* stands out as it contains 0.07% RA and is the only examined *Doodia* species containing RA. *Oceaniopteris gibba* was analysed twice: a fern from the Botanical Garden Marburg contained 0.21%, while another sample from the Palmengarten Frankfurt contained 0.45% 5‐CQA. The other analysed *Oceaniopteris* species, *O. ciliata*, has a 5‐CQA content of 0.17% and, furthermore, accumulates RA up to 0.11%. *N. brasiliense*, the only species in the *Neoblechnum* genus, had the most complex spectrum of phenolic compounds, with 0.60% 5‐CQA, 0.27% RA, and 0.68% BAB. *L. tabularis*, the only analysed species in the genus *Lomariocycas*, contained 0.11% 5‐CQA and 0.07% RA.

Further members of the subfamily Blechnoideae were not integrated into superclades. These were grouped into eight genera with ~38 species. Out of these genera, two were analysed: *Struthiopteris spicant* containing 0.68% and *Lomaria discolor* with 1.27% 5‐CQA.

*Subfamily Stenochlaenoideae*: Among the 16 species in three genera in the subfamily Stenochlaenoideae, *Stenochlaena tenuifolia* was analysed and contained 0.83% 5‐CQA.

*Subfamily Woodwardioideae*: The subfamily Woodwardioideae consists of 15 species in three genera, with *Woodwardia* being the most important, with 13 species. Five *Woodwardia* species were analysed. Their 5‐CQA content ranged from 0.41% in *W. radicans* to 0.79% in *W. prolifera*.

## DISCUSSION

There are few reports on the occurrence of specific phenolic compounds, such as 5‐CQA (**1**) and RA (**2**), in fern species; although fern species have been investigated for medicinal and nutritional uses due to the presence of health‐beneficial phenolics (Zeb & Ullah 2017; Tomou & Skaltsa 2018; Akhter *et al*. 2023; Kongsung *et al*. 2024; Salazar‐Chacón *et al*. 2024). We especially focused on the occurrence of caffeic acid esters, such as 5‐CQA and RA and their congeners, as well as their biosynthesis. For this, 32 fern species from the family Blechnaceae and all three subfamilies (Blechnoideae, Stenochlaenoideae, Woodwardioideae) were analysed.

### Caffeoyl‐5‐*O*‐quinic acid in Blechnaceae

5‐CQA (**1**) is widespread in land plants. Studies in ferns also show a broad occurrence of 5‐CQA (Dion *et al*. 2015; Zeb & Ullah 2017; Farràs *et al*. 2022; Salazar‐Chacón *et al*. 2024). Our study supported the above findings, since all extracts from species of the Blechnaceae contained 5‐CQA. This is not in full accordance with previous studies where, e.g., 5‐CQA, was not detected in *Blechnum polypodioides* or *Doodia caudata* (Petersen *et al*. 2009) or in the study of Bohm (1968), who analysed nine Blechnaceae species and did not detect 5‐CQA in *Sadleria cyatheoides, Sadleria pallida* (syn. *Sadleria hillebrandii*), *Woodwardia unigemmata*, *Woodwardia prolifera* (syn. *Woodwardia orientalis* var. *formosana*) or *Doodia dives*, although the latter three species were found to contain 5‐CQA in the present study. This might be related to less sensitive detection methods used in previous studies and masking by other substances of low concentrations of 5‐CQA. Furthermore, there may be differences in variants of the same fern species, as well as seasonal variations.

Taken together, our results suggest that 5‐CQA is abundant in Blechnaceae. Therefore, genes for the biosynthesis of 5‐CQA might have already been present in predecessors of the Blechnaceae.

### Rosmarinic acid in Blechnaceae

The occurrence of RA (**2**) is less common but not restricted to specific plant taxa (Petersen *et al*. 2009). Besides occurrence in hornworts (Takeda *et al*. 1990), RA has also been described in some fern species from different families (Pteridaceae, Salviniaceae, Athyriaceae; Andrade *et al*. 2016; Bohm 1968; Carballo‐Sanchez *et al*. 2022; Harborne 1966; Kongsung *et al*. 2024; Zeb & Ullah 2017). In this study, RA (**2**) was only detected in four of the 32 analysed species, suggesting that RA is much less abundant. Interestingly, the four RA‐containing species belong to four different genera (*Doodia*, *Oceaniopteris*, *Neoblechnum* and *Lomariocycas*), while other species of the same genera did not show RA accumulation in our study. All species containing RA are grouped in superclade B, as defined by de Gasper *et al*. (2017). In a previous study, RA was found in *Oceaniopteris gibba* (syn. *B. gibbum*; Abdullah 2010; Petersen *et al*. 2009). This could not be reproduced, neither in plant material from the same botanical garden (Marburg) nor material from a different source (Palmengarten Frankfurt). In the study by Abdullah (2010) detection of RA was only performed by HPLC, with no further LC/MS verification. Here, the amount of RA in consecutive years differed highly (0.07% and 0.43%), indicating that the accumulation of phenolic compounds may be influenced by environmental conditions. Bohm (1968) analysed 40 fern species from various families using thin‐layer chromatography, but detected RA only in one species, *N. brasiliense* (syn. *B. brasiliense*).

### Blechnic acid B (**7**) in *Neoblechnum brasiliense*

The novel compound with a prominent peak in HPLC chromatograms was isolated and its structure elucidated using LC/ESI‐MS and LC/ESI‐MS/MS data, as well as NMR. The compound was named blechnic acid B (BAB, **7**) since its core structure is blechnic acid (Bohm 1968; Wada *et al*. 1992). BAB is a dimer of caffeoyl‐4′‐hydroxyphenyllactic acid (isorinic acid). The same compound was previously isolated from *Orthosiphon aristatus*, but without full stereochemical information (compound 20 in the publication of Sumaryono *et al*. 1991). Blechnic acid B was also proposed to be present in *S. miltiorrhiza* (3″‐deoxy‐3″′‐deoxy‐salvianolic acid B; Cao *et al*. 2018) by comparing fragmentation data (as done here) with the more hydroxylated salvianolic acid B, which is an already known compound in *S. miltiorrhiza*.

As described for blechnic acid and salvianolic acid, also BAB (**7**) and its derivatives might have interesting pharmacological properties. An intraperitoneal dose of 30 mg kg^−1^ salvianolic acid B in mice with experimental autoimmune encephalomyelitis (EAE) found an effective decline of disease severity by inhibition of T_h_1 cell response and inhibition of infiltration of inflammatory cells into the central nervous system (Dong *et al*. 2016). An dose of 10 or 20 mg kg^−1^ salvianolic acid B in mice transplanted with MC38 colon cancer cells showed a tumour suppression rate of 43.4% and 63.2%, respectively, by inhibiting the ubiquitin carboxy‐terminal hydrolase 2 (USP2) and, ultimately, downregulation of programmed death‐ligand 1 (PD‐L1), thus enhancing the killing activity of T cells (Kuang *et al*. 2023). Other effects of salvianolic acid B have been summarized by He *et al*. (2023). Hence, BAB could have similar effects as salvianolic acid B because of the resemblance in chemical structure.

A biosynthetic pathway for blechnic acid was proposed by Wang *et al*. (2001): two caffeic acid molecules are oxidatively coupled and the participation of a dirigent protein suggested. BAB (**7**) could be synthesized by a similar mechanism using two caffeoyl‐4′‐hydroxyphenyllactic acid radicals instead of caffeic acid (see Fig. S2). Although only suggested by fragmentation data, 3′‐hydroxyblechnic acid B, 3″′‐hydroxyblechnic acid B (**3**, **6**), and 3′,3″′‐dihydroxyblechnic acid (**4**) are also present in the extract of *N. brasiliense*. This suggests that the proposed dirigent protein could either accept both caffeoyl‐4′‐hydroxyphenyllactic acid (**5**) and RA (**2**), or that there is more than one dirigent protein forming blechnic acid B (**7**) and its more hydroxylated derivatives (**3**, **4**, and **6**).

Another possible biosynthetic pathway would be that blechnic acid is first formed and then acylated. In this case, further enzymes would be required: two special blechnic acid‐CoA ligases must activate the two different carboxyl groups (of which one is sterically hindered) to CoA thioesters. Two distinct BAHD acyltransferases would then transfer the CoA‐activated blechnic acid to 4‐hydroxyphenyllactic acid and/or 3,4‐dihydroxyphenyllactic acid to produce blechnic acid B and its derivatives. To our knowledge, no CoA ligases or BAHD acyltransferases are known to accept a substrate as large as blechnic acid. We therefore consider it unlikely that blechnic acid is formed first and then acylated and propose that the putative dirigent proteins form the lignans directly from isorinic/rosmarinic acids.

### Phylogeny of Blechnaceae and occurrence of rosmarinic acid, caffeoyl‐5‐*O*‐quinic acid, and blechnic acid B

**Fig. 4:**
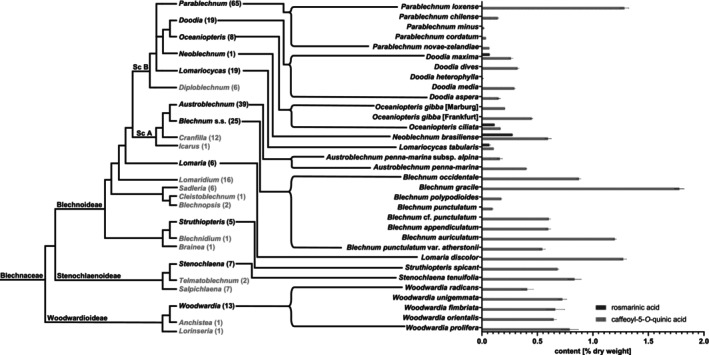
Phylogenetic relationships and content of phenolic compounds in Blechnaceae. Left: Phylogenetic tree of Blechnaceae based on plastid DNA (redrawn from Gasper *et al*. (2017); scale not adopted), Middle: Genera of Blechnaceae with species count in brackets (Gasper *et al*. 2016), genera in grey that have not been analysed, Right: Occurrence and content of rosmarinic acid and caffeoyl‐5‐*O*‐quinic acid in the analysed species (this study); Sc A, Superclade A; Sc B, Superclade B.

The phylogenetic tree established by de Gasper *et al*. (2017) clearly resolves the three subfamilies of family Blechnaceae, and within subfamily Blechnoideae the division into superclades A and B, as well as genera not belonging to a superclade (Fig. 4). In combination with our data on the occurrence of 5‐CQA (**1**), RA (**2**), and BAB (**7**), it becomes clear that the presence of 5‐CQA in all investigated species does not lead to discrimination of the two superclades. RA and BAB, on the other hand, could only be found in superclade B. However, it should be noted that RA has additionally been detected in ferns outside the Blechnaceae, namely members of fern families Pteridaceae (Zeb & Ullah 2017), Salviniaceae (Carballo‐Sanchez *et al*. 2022), and Athyriaceae (Kongsung *et al*. 2024). Further investigations into the specialized metabolism of ferns could lead to resolution of more widespread presence of RA and related phenolic compounds in this plant group.

## AUTHOR CONTRIBUTIONS

The study was planned by both authors. All experimental procedures were carried out by MU. Both authors collaborated in analysing data and writing the manuscript.

## CONFLICT OF INTEREST STATEMENT

The authors have no competing interests to declare that are relevant to the content of this article.

## Supporting information


**Fig. S1.** HPLC analysis of the ester hydrolysis of blechnic acid B (**7**) using chiral chromatography. **(a)** 100 nmol racemic 4‐hydroxyphenyllactic acid standard, **(b)** 50 nmol (*s*)‐4‐hydroxyphenyllactic acid standard **(c)** ester hydrolysis of blechnic acid B **(d)** 100 nmol racemic 4‐hydroxyphenyllactic acid standard + ester hydrolysis of blechnic acid B.
**Fig. S2.** Proposed biosynthesis of blechnic acid B (**7**) in *Neoblechnum brasiliense*.
**Table S1.** Species of the Blechnaceae family used for extraction of phenolic compounds. The source botanical gardens are the following: BGBa = Ökologisch‐Botanischer Garten Universität Bayreuth (Germany), BGB = Botanischer Garten Freie Universität Berlin (Germany), BGHa = Botanischer Garten Universität Hamburg (Germany), BGHe = Botanischer Garten Ruprecht‐Karls‐Universität Heidelberg (Germany), BGMa = Botanischer Garten Philipps‐Universität Marburg (Germany), BGMu = Botanischer Garten München‐Nymphenburg (Germany), BGT = Botanischer Garten Eberhard‐Karls‐Universität Tübingen (Germany), MP = collected near Marburg and identified by Maike Petersen, PGM = Palmengarten Frankfurt/Main (Germany); ^1)^ according to Field (2020).
**Table S2.** ^1^H‐NMR and ^13^C‐NMR spectroscopic data of isolated blechnic acid B (**7**) from *Neoblechnum brasiliense*. The systems 1′–9′ and 1″–9″ are interchangeable.
