# Supplementary material for: Phenolic compounds in species of the Blechnaceae
Source: Plant Biol (Stuttg). 2025 Sep 28;28(1):282–91. doi: 10.1111/plb.70116 (PMC12710811; doi:10.1111/plb.70116)
Supplement: Supplementary file 1 — Fig. S1. HPLC analysis of the ester hydrolysis of blechnic acid B (7) using chiral chromatography. (a) 100 nmol racemic 4‐hydroxyphenyllactic acid standard, (b) 50 nmol (s)‐4‐hydroxyphenyllactic acid standard (c) ester hydrolysis of blechnic acid B (d) 100 nmol racemic 4‐hydroxyphenyllactic acid standard + ester hydrolysis of blechnic acid B. Fig. S2. Proposed biosynthesis of blechnic acid B (7) in Neoblechnum brasiliense. Table S1. Species of the Blechnaceae family used for extraction of phenolic compounds. The source botanical gardens are the following: BGBa = Ökologisch‐Botanischer Garten Universität Bayreuth (Germany), BGB = Botanischer Garten Freie Universität Berlin (Germany), BGHa = Botanischer Garten Universität Hamburg (Germany), BGHe = Botanischer Garten Ruprecht‐Karls‐Universität Heidelberg (Germany), BGMa = Botanischer Garten Philipps‐Universität Marburg (Germany), BGMu = Botanischer Garten München‐Nymphenburg (Germany), BGT = Botanischer Garten Eberhard‐Karls‐Universität Tübingen (Germany), MP = collected near Marburg and identified by Maike Petersen, PGM = Palmengarten Frankfurt/Main (Germany); 1) according to Field (2020). Table S2. 1H‐NMR and 13C‐NMR spectroscopic data of isolated blechnic acid B (7) from Neoblechnum brasiliense. The systems 1′–9′ and 1″–9″ are interchangeable. [file PLB-28-282-s001.docx]

**Supporting Information for**

**Phenolic compounds in species of the Blechnaceae**

Maximilian Ufland and Maike Petersen*

Institut für Pharmazeutische Biologie und Biotechnologie, Philipps-Universität Marburg, Robert-Koch-Str. 4, 35037 Marburg, Germany

**Supporting Figures**

**Fig. S1.** HPLC analysis of the ester hydrolysis of blechnic acid B (**7**) using chiral chromatography. **(a)** 100 nmol racemic 4-hydroxyphenyllactic acid standard, **(b)** 50 nmol (*S*)-4-hydroxyphenyllactic acid standard **(c)** ester hydrolysis of blechnic acid B **(d)** 100 nmol racemic 4-hydroxyphenyllactic acid standard + ester hydrolysis of blechnic acid B.

**Fig. S2.** Proposed biosynthesis of blechnic acid B (**7**) in *Neoblechnum brasiliense*.

**Supporting Tables**

**Table S1.** Species of the Blechnaceae family used for extraction of phenolic compounds. The source botanical gardens are the following: BGBa = Ökologisch-Botanischer Garten Universität Bayreuth (Germany), BGB = Botanischer Garten Freie Universität Berlin (Germany), BGHa = Botanischer Garten Universität Hamburg (Germany), BGHe = Botanischer Garten Ruprecht-Karls-Universität Heidelberg (Germany), BGMa = Botanischer Garten Philipps-Universität Marburg (Germany), BGMu = Botanischer Garten München-Nymphenburg (Germany), BGT = Botanischer Garten Eberhard-Karls-Universität Tübingen (Germany), MP = collected near Marburg and identified by Maike Petersen, PGM = Palmengarten Frankfurt/Main (Germany); ^1)^ according to Field (2020).

| **Species name according to source botanical garden** | **Species name according to Gasper *et al.* (2016)** | **Source** | **Accession** |
| --- | --- | --- | --- |
| **Blechnoideae – superclade B** | | | |
| *Blechnum loxense* (Kunth) Hook. Ex Salomon | *Parablechnum loxense* (Kunth) Gasper & Salino | BGBa | 981308 |
| *Blechnum chilense* (Kaulf.) Mett. | *Parablechnum chilense* (Kaulf.) Gasper & Salino | BGHa | 2013-F-176 |
| *Blechnum minus* (R.Br.) Allan | *Parablechnum minus* (R.Br.) Gasper & Salino | BGMu | 1995/3206 |
| *Blechnum cordatum* (Desv.) Hieron. | *Parablechnum cordatum* (Desv.) Gasper & Salino | PFM | 14-27564-5 |
| *Blechnum novae-zelandiae* T.C.Chambers & P.A.Farrant | *Parablechnum novae-zelandiae* (T.C.Chambers & P.A.Farrant) Gasper & Salino | PFM | 12-26292-5 |
| *Doodia maxima* R.Br. ex C.Chr. | *Doodia maxima* J.Sm. ex C.Chr. | BGT | 10022 |
| *Blechnum dives* (Kunze) Christenh. | *Doodia dives* Kunze | BGT | 5026 |
| *Blechnum doodianum* Christenh. | *Doodia heterophylla* (F.M.Bailey) Domin | BGMu | S/0002 |
| *Blechnum medium* (R.Br.) Christenh. | *Doodia media* R.Br. | PFM | 19-30916-5 |
| *Blechnum neohollandicum* Christenh. | *Doodia aspera* R.Br. | BGHe | 106399 |
| *Blechnum gibbum* (Labill.) Mett. | *Oceaniopteris gibba* (Labill.) Gasper & Salino | BGMa | 1995/340 |
| *Blechnum gibbum* (Labill.) Mett. | *Oceaniopteris gibba* (Labill.) Gasper & Salino | PFM | 7-23298-0 |
| *Blechnum moorei* C. Chr. | *Oceaniopteris ciliata* (T.Moore) Gasper & Salino | BGBa | 20268 |
| *Blechnum brasiliense* Desv. | *Neoblechnum brasiliense* (Desv.) Gasper & V.A.O.Dittrich | BGMa | 1977/388 |
| *Blechnum tabulare* (Thunb.) Kuhn | *Lomariocycas tabularis* (Thunb.) Gasper & A.R.Sm. | BGB | 17557483 |
| **Blechnoideae – superclade A** | | | |
| *Blechnum penna-marina* (Poiret) Kuhn subsp. *alpina* (R.Br.) T.C.Chambers & P.A.Farrant | *Austroblechnum penna-marina* subsp. *alpina* (R.Br.) A.R.Field^1)^ | BGMu | 2005/2028 |
| *Blechnum penna-marina* (Poir.) Kuhn | *Austroblechnum penna-marina* (Poir.) Gasper & V.A.O.Dittrich | BGB | 22717480 |
| *Blechnum occidentale* L. | *Blechnum occidentale* L. | BGMa | 1994/818 |
| *Blechnum gracile* Kaulf. | *Blechnum gracile* Kaulf. | BGMa | 2005/3 |
| *Blechnum polypodioides* Raddi | *Blechnum polypodioides* Raddi | BGMa | 2003/18 |
| *Blechnum punctulatum* Sw. var. *punctulatum* | *Blechnum punctulatum* Sw. | BGT | 1828 |
| *Blechnum* cf. *punctulatum* Sw. | *Blechnum* cf. *punctulatum* Sw. | BGBa | 41130 |
| *Blechnum appendiculatum* Willd. | *Blechnum appendiculatum* Willd. | BGB | 141541220 |
| *Blechnum australe* subsp. *auriculatum* (Cav.) de la Sota | *Blechnum auriculatum* Cav. | BGB | 57139570 |
| *Blechnum punctulatum* var. *atherstonei* (Pappe & Rawson) Sim | *Blechnum punctulatum* Sw. var. *atherstonii* R.Sim | BGT | 1434 |
| **Blechnoideae – further genera** | | | |
| *Blechnum discolor* (G.Forst.) Keyserl. | *Lomaria discolor* (G.Forst.) Willd. | PFM | 12-26398-5 |
| *Struthiopteris spicant* (L.) F.W.Weiss | *Struthiopteris spicant* (L.) F.W.Weiss | MP | - |
| **Stenochlaenoideae** | | | |
| *Stenochlaena tenuifolia* (Desv.) T. Moore | *Stenochlaena tenuifolia* (Desv.) T. Moore | BGT | 15201 |
| **Woodwardioideae** | | | |
| *Woodwardia radicans* (L.) Sm. | *Woodwardia radicans* (L.) Sm. | BGT | 7176 |
| *Woodwardia unigemmata* (Makino) Nakai | *Woodwardia unigemmata* (Makino) Nakai | BGHe | 108857 |
| *Woodwardia fimbriata* Sm. | *Woodwardia fimbriata* Sm. | BGHa | 2021-F-52 |
| *Woodwardia orientalis* Sw. | *Woodwardia orientalis* Sw. | BGT | 5033 |
| *Woodwardia prolifera* Hook. & Arn. | *Woodwardia prolifera* Hook. & Arn. | BGT | 11450 |

**Table S2.** ^1^H-NMR and ^13^C-NMR spectroscopic data of isolated blechnic acid B (**7**) from *Neoblechnum brasiliense*. The systems 1’-9’ and 1’’’-9’’’ are interchangeable.

| Atom Nr. | δ_H_ [ppm] | M (J) | δ_C_ [ppm] |
| --- | --- | --- | --- |
| 1 | - | - | 124.53 |
| 2 | - | - | 126.52 |
| 3 | - | - | 149.21 |
| 4 | - | - | 145.19 |
| 5 | 6.85 | d (8.4 Hz) | 118.27 |
| 6 | 7.13 | d (8.5 Hz) | 122.36 |
| 7 | 7.46 | d (16.0 Hz) | 143.3 |
| 8 | 6.14 | d (16.0 Hz) | 116.67 |
| 9 | - | - | 167.92 |
| 1’ | - | - | 128.64 |
| 2’ | 7.12 | d (8.5 Hz) | 131.59 |
| 3’ | 6.69 - 6.73 | m | 116.28 |
| 4’ | - | - | 157.35 |
| 5’ | 6.69 - 6.73 | m | 116.28 |
| 6’ | 7.12 | d (8.5 Hz) | 131.59 |
| 7’ | 3.04; 3.12 | m | 37.67 |
| 8’ | 5.17 | dd (7.6 Hz; 3.5 Hz) | 75.54 |
| 9’ | - | - | 172.66 *or* 173.69 |
| 1’’ | - | - | 133.63 |
| 2’’ | 6.78 | ds (2.1 Hz) | 113.4 |
| 3’’ | - | - | 146.8 |
| 4’’ | - | - | 146.64 |
| 5’’ | 6.75 | d (8.1 Hz) | 116.38 |
| 6’’ | 6.68 | dd (8.2 Hz; 2.2 Hz) | 118.37 |
| 7’’ | 5.9 | d (5.0 Hz) | 88.4 |
| 8’’ | 4.34 | d (5.0 Hz) | 58.25 |
| 9’’ | - | - | 172.1 |
| 1’’’ | - | - | 128.2 |
| 2’’’ | 6.69 - 6.73 | m | 131.24 |
| 3’’’ | 6.53 | d (8.5 Hz) | 116.2 |
| 4’’’ | - | - | 157.08 |
| 5’’’ | 6.53 | d (8.5 Hz) | 116.2 |
| 6’’’ | 6.69 - 6.73 | m | 131.24 |
| 7’’’ | 2.87; 3.01 | m | 37.21 |
| 8’’’ | 5.15 | dd (9.5 Hz; 3.5 Hz) | 74.8 |
| 9’’’ | - | - | 172.66 *or* 173.69 |

**References**

Field A.R. (2020) Classification and typification of Australian lycophytes and ferns based on Pteridophyte Phylogeny Group classification PPG I. *Australian Systematic Botany*, **33**, 1.

Gasper A.L. de, Dittrich Vinicius Antonio de Oliveira, Smith A.R., Salino A. (2016) A classification for Blechnaceae (Polypodiales: Polypodiopsida): New genera, resurrected names, and combinations. *Phytotaxa*, **275**, 191–227.
